# Exercise-Induced Central Fatigue: Biomarkers and Non-Medicinal Interventions

**DOI:** 10.14336/AD.2024.0567

**Published:** 2025-06-27

**Authors:** Ying Yang, Zhi Feng, Yu-hang Luo, Jue-miao Chen, Yu Zhang, Yi-jun Liao, Hui Jiang, Yinxi Long, Bo Wei

**Affiliations:** ^1^Institute of Translational Medicine, School of Basic Medical, Department of Special Medicine, School of Public Health, Hengyang Medical College, University of South China, Hengyang, 421001, China.; ^2^Department of Neurology, Affiliated Hengyang Hospital of Hunan Normal University & Hengyang Central Hospital, Hengyang, 421001, China.

**Keywords:** exercise-induced central fatigue, biomarkers, neurotransmitters, caffeine, amino acids, carbohydrates

## Abstract

Fatigue, commonly experienced in daily life, is a feeling of extreme tiredness, shortage or lack of energy, exhaustion, and difﬁculty in performing voluntary tasks. Central fatigue, defined as a progressive failure to voluntarily activate the muscle, is typically linked to moderate- or light-intensity exercise. However, in some instances, high-intensity exercise can also trigger the onset of central fatigue. Exercise-induced central fatigue often precedes the decline in physical performance in well-trained athletes. This leads to a reduction in nerve impulses, decreased neuronal excitability, and an imbalance in brain homeostasis, all of which can adversely impact an athlete's performance and the longevity of their sports career. Therefore, implementing strategies to delay the onset of exercise-induced central fatigue is vital for enhancing athletic performance and safeguarding athletes from the debilitating effects of fatigue. In this review, we discuss the structural basis, measurement methods, and biomarkers of exercise-induced central fatigue. Furthermore, we propose non-pharmacological interventions to mitigate its effects, which can potentially foster improvements in athletes' performances in a healthful and sustainable manner.

## 1. Introduction

Fatigue is a sensation an emotion resulting from the interaction of the brain and muscles, arising from altered function during exercise. It acts as a key regulator to maintain homeostasis across all bodily systems, ensuring protection from injury post-exercise [1]. Fatigue can stem from various processes along the motor pathway, which are broadly categorized into central and peripheral origins. Peripheral fatigue is characterized by a decrease in twitch or tetanic force due to peripheral nerve stimulation in a relaxed muscle. This type of fatigue arises from changes at or beyond the neuromuscular junction and is often assessed through pre/post-exercise reductions in quadriceps twitch-force [2]. Central fatigue, emanating from the central nervous system, is described as a progressive inability to voluntarily activate muscles. It is typically assessed by stimulating the peripheral motor nerve during an isometric maximum voluntary contraction [3]. The contributions of peripheral and central fatigue to reduced exercise capacity vary based on exercise duration, intensity, gender, exercise type, and psychological and environmental factors [4].

A prevailing notion suggests that peripheral fatigue correlates with short-duration, high-intensity exercises, while central fatigue is more evident during resistance exercises of moderate to light intensity [5-7]. Nonetheless, some researchers argue that central fatigue might modulate peripheral fatigue, impacting exercise capacity even during high-intensity routines [8]. Further studies indicated that certain central factors might control peripheral fatigue to a crucial threshold, preventing reduced exercise capacity during knee extensor exercises in older men [2]. The interplay between peripheral and central fatigue largely relies on group III/IV muscle afferents within muscles [9].

Excessive exercise intensity leading to fatigue can result in athletes experiencing nerve disorders, muscle movement abnormalities, and endocrine imbalances, jeopardizing their performance and career longevity. Yet, Chen et al. noted that central fatigue, marked by a drop in nerve impulses, neuronal excitability, and brain homeostasis imbalance, manifests before any decline in physical performance in adept athletes [10]. Proper training can also enhance athletes' cortical excitability and response rates by influencing specific brain regions, thereby elevating the central fatigue threshold and preparing them for sports challenges [11, 12]. As such, a thorough comprehension of relevant biomarkers and strategic interventions to deter central fatigue onset is paramount. This not only safeguards the body's normal structure and function, particularly the brain but also offers valuable insights for athlete training.

## 2. Structural basis of exercise-induced central fatigue

Motor neuron movement is orchestrated by classical motor control regions (including the prefrontal cortex, motor cortex, basal ganglia, and cerebellum) within the CNS and by feedback signals from muscles [13]. In particular, the interaction between the dorsolateral prefrontal cortex (PFC) and the insular cortex, anterior cingulate cortex, ventromedial and lateral prefrontal cortex, as well as the feedback effect of the layer I spinothalamic pathway, vagus nerve, and glossopharyngeal medullary thalamic pathway are crucial. Additionally, the dopaminergic mesolimbic and locus ceruleus-norepinephrine pathways play a significant role. Some research suggests that the activation of both subcortical and cortical structures implies the PFC's role in modulating exercise tolerance and performance [14]. Functional MRI analyses have delineated the sequence whereby increasing exercise intensity can activate or deactivate various brain regions, encompassing subcortical areas. Beyond activating classical motor control regions, regions tied to autonomic regulation (e.g., the insular cortex) are engaged, whereas cognitive-related regions (like the PFC) see diminished activation [15]. Interestingly, the motor cortex remains active throughout the exercise regimen, while the cerebellum is activated only during low-intensity efforts and not high-intensity sessions [16].

Thinly myelinated (group III) and unmyelinated (group IV) nerve fibers present in skeletal muscle play a pivotal role in the interplay between central and peripheral fatigue. Once these receptors are triggered, the discharge frequency of group III/IV muscle afferents dynamically adjusts via the dorsal horn of the spinal cord. Once activated, these receptors adjust the discharge frequency of group III/IV muscle afferents through the dorsal horn of the spinal cord. This results in modifications in feedback to the CNS, touching on areas related to central fatigue (e.g., the motor cortex, α-motoneurons, cingulate, and insular cortex) [17].It's widely believed that feedback from group III/IV muscle afferents exerts a dual influence on the CNS, contingent on the presence or absence of fatigue. In situations where there is no fatigue, feedback from these afferents tends to inhibit spinal motoneurons and activate cortical cells. Conversely, during intense exercise, feedback from the same afferents can suppress the motor cortex and instigate exercise-induced central fatigue [18]. In summary, in the absence of fatigue, muscle afferent nerves from the group III/IV facilitate the activation of the motor cortex. Conversely, when fatigue is present, muscle afferents from the group III/IV impede the functioning of the motor cortex [19]. Central fatigue might also modulate peripheral fatigue to a vital threshold via group III/IV muscle afferents [2].

However, there's a debate surrounding the effects of group III/IV afferents on motor cortical networks and their contribution to diminishing voluntary activation (an indicator of central fatigue). Notably, motoneuron excitability isn't diminished during fatiguing maximal isometric contractions of the elbow flexor muscle. Instead, motor cortical output cell excitability is augmented and then returns to baseline values shortly post-contraction [20]. Further inquiries are necessary to elucidate how the CNS behaves during fatigue.

## 3. The measurement of exercise-induced central fatigue

Central fatigue is elusive, making empirical evidence of its existence challenging to procure. The quantification of exercise-induced changes in cortical neuronal excitability, closely associated with central fatigue, can be achieved through the utilization of non-invasive stimulation techniques. The inaugural method identified for assessing central fatigue is the interpolated twitch technique. Its amplitude is thought to reflect the fraction of untapped muscle force generation potential during a voluntary contraction, subsequently providing insight into the degree of voluntary muscle drive. An elevation in the amplitude of interpolated twitch caused by exercise during maximal efforts is frequently cited as evidence of a diminishing voluntary drive, which is indicative of central fatigue [21, 22]. In short, if the electrically evoked maximal twitch (SIT) is superimposed on the maximal voluntary isometric contraction (MVC), then the fatigue-induced increase in SIT amplitude may be caused by central fatigue, suggesting a decrease in the ability of the cerebral motor cortex to drive the muscle [23]. Nonetheless, some researchers argue that the interpolated twitch might fall short in accurately gauging voluntary drive, as it doesn't adequately factor in established peripheral contributors to muscle fatigue, including impaired membrane excitability, decreased muscle fiber strength, and slowed contraction caused by fatigue [24, 25]. Transcranial magnetic stimulation (TMS), when applied to the contralateral hemisphere of the motor cortex, induces a short-latency electromyography response called motor evoked potential (MEP) in the target muscle. However, TMS alone cannot distinguish fatigue-related changes in the excitability of the motor cortex, spinal motor neurons or muscle fibers, so it is also necessary to elicit cervical spinal motor evoked potential (CMEP) and maximum compound muscle action potential (Mmax) [26]. Consequently, a model has been developed that computes the force a muscle produces during voluntary isometric contractions compared to electrical stimulation. This suggests that peripheral factors alone can account for the force dynamics typically ascribed to central fatigue [27]. However, tools like near-infrared spectroscopy, electroencephalography, and functional MRI permit the direct probing of brain activity during exercise, underscoring the role of central fatigue in modulating exercise performance [16]. Thus, there is a pressing need for advanced, precise technologies to assess brain function during exercise.

## 4. Common Biomarkers of exercise-induced central fatigue

### 4.1. Neurotransmitters

Neurotransmitters implicated in exercise-induced central fatigue primarily encompass serotonin (5-hydroxytryptamine; 5-HT), dopamine (DA), noradrenaline (NA), gamma-aminobutyric acid (GABA), and glutamate (Glu). These may underpin the central protective inhibition mechanism [28].

#### 4.1.1. 5-HT and Its Associated Regulators

5-HT, an inhibitory neurotransmitter, derives from the amino acid tryptophan (TRP) that is shuttled across the blood-brain barrier from plasma. With elevated free TRP levels in sample of blood, leading to some of the central nervous system neurons in the 5-HT increased, change the neural electrophysiological activities, which control the movement of the brain activities, may lead to a central fatigue. TRP hydroxylase (TPH) governs the rate of 5-HT synthesis in serotonergic neurons located within the raphe nuclei [29]. An experiment with male Wistar rats injected with L-TRP compared to controls revealed levels of 5-HT in various brain regions like the preoptic area (POA), hypothalamus, frontal cortex, and hippocampus. In the POA, a decrease in 5-HT levels was associated with the onset of fatigue, supporting the idea that 5-HT levels play a crucial role in central fatigue induced by exercise. Higher levels of 5-HT in the brain appear to accelerate fatigue onset [30]. Human in vivo research further substantiates that 5-HT exerted on motoneurons is instrumental in central fatigue [31]. Claghorn and colleagues also discerned that 5-HT-mediated central fatigue primarily contributes to enhanced endurance capacity in mice [32]. Additionally, 5-HT is vital for balanced neurotransmission, ensuring optimal neuromodulation and neuronal network adjustments [33, 34]. Central neuromodular disturbances triggered by exhaustive exercise can be rectified by the serotonergic system, potentially mitigating or staving off transient psychological disturbances in overtrained athletes [34]. Further studies spotlighting 5-HT's roles suggest that sustained low-intensity serotonergic neurotransmission primarily influences supraspinal fatigue processes, rather than directly impacting motor system output during submaximal exercises [35].

Serotonergic neurons reside within the CNS's raphe nuclei. Extracellular 5-HT has been shown to activate 5-HT receptors, modulating central fatigue during exercise. Extended stimulation of the raphe-spinal pathway can elevate 5-HT to levels potent enough to engage 5-HT1A or 5-HT1B receptors at motoneuron's axon initial segments [36]. Enhanced activation of 5-HT1A receptors spurs 5-HT synthesis during treadmill exercises in stress-afflicted rats, thereby hastening central fatigue onset [37]. Some studies verify that while 5-HT1A receptor activation impedes mesolimbic DA neurotransmission, 5-HT1B receptor activation can restrain local 5-HT synthesis and release while amplifying DA activity [38]. Polysaccharide from Spirulina platensis (PSP) inhibits exercise-induced 5-HT synthesis and TPH2 expression and increases the expression of 5-HT1B in the caudate putamen of exercise rats [39]. Calamus can suppress the 5-HT induced by motion, TPH2 mRNA and protein expression, prevent exercise-induced the midbrain dorsal seam 5-HT1B mRNA and protein expression. In this regard, caffeine has the same effect [40]. However, increased serotonin synthesis does not necessarily increase serotonin release, and brain function is not determined by a single neurotransmitter. Therefore, the serotonin/dopamine ratio is considered to be a more accurate parameter for central fatigue [41]. Besides, predominantly, the serotonergic system exerts an inhibitory influence over DA activity [42].

#### 4.1.2. DA and NA

DA and NA are pivotal neurotransmitters derived from the amino acid tyrosine. They're linked to the "central" facet of fatigue due to their renowned influence on motivation and motor behavior. The central dopaminergic system modulates muscle tension, priming the body for action under the guidance of the cerebral cortex [43]. Furthermore, diminished DA stimulation within the cerebral cortex during exercise might instigate central fatigue [4]. Some research suggests that DA and NA in the POA and anterior hypothalamus could influence thermal regulation. DA is noted for its ergogenic effects, bolstering heat storage capacity and enhancing hyperthermia tolerance [42]. Locus coeruleus (LC) and the brainstem noradrenergic groups A1 are involved in the mechanism of skin heat loss during exercise. The LC nucleus may regulate caudal sympathetic tone and integrate the central network LC/POA, which may be an important circuit for temperature regulation during exercise [44].Under heated conditions, DA elevates exercise performance and forestalls fatigue, whereas NA reuptake can hamper performance [45, 46].

#### 4.1.3. GABA and Glu

GABA stands as a principal inhibitory neurotransmitter in the CNS. Direct associations between GABA and central fatigue remain unconfirmed. Given GABA's influence on the immune system, it's hypothesized that it might also play a role in fatigue's onset [47]. Notably, thalamic GABA concentrations in healthy individuals surge following a 12-week regimen of yoga or walking [48]. The administration of plant-derived GABA notably extends the loaded-swimming duration in mice, underscoring GABA's anti-fatigue capabilities [49].

Glu, a conditionally essential amino acid frequently employed in sports nutrition, has connections to central fatigue. Elevated Glu levels in the cerebellum and hippocampus, coupled with an uptick in the Glu-Gln (glutamine) cycle in the brain, are intimately tied to central fatigue [50]. The imbalance of Glu-Gln cycle may be an important factor in exercise-induced central fatigue in rats tested from acute endurance to post-exhaustion [51]. A review posits that Glu can delay fatigue by bolstering the Krebs cycle anaplerosis and gluconeogenesis, encouraging glycogen synthase activity, and serving as a non-toxic ammonia conduit to prevent accumulation [52]. Explorations into the link between subthalamic nucleus activity alterations and exercise-induced central fatigue suggest that dynamic shifts in subthalamic nucleus activity are intrinsically linked with early fatigue onset. This might be partly attributed to the Glu/GABA ratio in the subthalamic nucleus's extracellular fluid [53].

### 4.2. Biomarkers of Oxidative and Nitrosative Stress in the Brain

Key players in oxidative stress primarily encompass malondialdehyde (MDA, a lipid oxidation byproduct) and three antioxidant enzymes: superoxide dismutase (SOD), glutathione peroxidase (GSH-Px), and catalase (CAT). Superoxide radicals can metamorphose into hydrogen peroxide, which SOD and GPx and/or CAT then process into water [54]. Diverse hypoxic exercise methods elevate the activities of SOD, GSH-Px, and CAT, as well as MDA content in mouse brains. Yet, exercise under hypoxic conditions exacerbates brain oxidative damage, particularly post-exhaustive sessions, even as it amplifies free radical production [55]. In addition, Nrf2 is one of the important transcription factors regulating oxidative stress reaction. Therefore, it is of application value to develop natural antioxidants that can regulate Nrf2-related signaling pathways to improve exercise-induced central fatigue [56].Proper exercise is paramount for enhancing rat brain antioxidant capacities. Moreover, antioxidant supplements exhibit pronounced pharmacological benefits in mitigating fatigue [57, 58].

Nitric oxide (NO), a prevalent reactive nitrogen species, is widely studied as a factor enhancing blood flow. Nitric oxide synthase (NOS), responsible for regulating NO production, is found in cerebrovascular endothelial and nerve cells, especially in the cerebral cortex and hypothalamus. As the key regulator of blood flow, any slight alteration in NO levels can influence physical performance and, in turn, affect central or peripheral fatigue [59]. Acutely obstructing central nNOS curtails rat running performance during treadmill activities, underlining the potential influence of central nNOS and NO on exercise-induced central fatigue [60]. Furthermore, NO signaling in the paraventricular nucleus and dorsomedial hypothalamus, stimulated by nNOS induction, plays a crucial role in enhancing exercise capacity [61].

### 4.3. Brain glycogen and lactate

The primary brain energy reserve is glycogen—originating from glucose housed in astrocytes—which astrocytes then convert into lactate, a neuronal energy substrate [62]. Astrocytes release lactate in response to neuronal activation, and neurons absorb it, oxidizing it to pyruvate for acetyl-CoA synthesis in the tricarboxylic acid cycle [63].

During extended exercise sessions, blood glucose concentrations tend to diminish. As blood glucose depletes, brain glycogen also dwindles during prolonged activities. Investigations have pinpointed correlations between declining cortical cerebrovascular glycogen levels during long exercise and the activation of monoamine metabolism—a plausible central fatigue contributor [64]. Direct glucose infusion into the hippocampus of rats undergoing intense treadmill runs can counteract the 5-HT surge, modulating 5-HT release/reuptake rather than its synthesis to alleviate central fatigue [65]. Recent findings suggest astrocytic glycogen-derived lactate as an essential energy source in the brain to sustain endurance during exhaustive workouts. Blocking glycogenolysis and lactate transport reduces lactate production in the brain and lowers ATP levels in the hippocampus during exhaustion, demonstrating that brain ATP derived from lactate and other energy sources is more critical than muscle ATP [66]. Additionally, lactate might forestall fatigue onset by amplifying primary motor cortex excitability [67]. However, studies have also shown that increased L-lactate levels in the brain exacerbate central fatigue during exercise by activating the brain lactate receptor, also known as hydroxy-carboxylic acid receptor 1 (GPR81), and the exact mechanism remains unclear [68]. We summarize the role of biomarkers associated with exercise-induced central fatigue (Table 1).

**Table 1:** Effects of biomarkers related to exercise-induced central fatigue.

Biomarkers	Effects
**5-HT**	Correcting the neuromodular disturbance in an exhaustive state [34], inhibiting the dopaminergic systems [42], and promoting exercise-induced central fatigue [69]
**TPH**	Catalyzing the synthesis of 5-HT
**5-HT_1A_**	Suppressing DA neurotransmission and facilitating 5-HT synthesis to promote exercise-induced central fatigue [37, 38]
**5-HT_1B_**	Inhibiting local synthesis and the release of 5-HT, as well as increasing DA activity [38, 42]
**DA**	Improving tolerance in heat stress, alleviating exercise-induced central fatigue, and promoting exercise performance [42, 45, 46]
**NA**	Promoting exercise-induced central fatigue and decreasing performance [45, 46]
**GABA**	Inhibiting exercise-induced central fatigue and improving performance [49]
**Glu**	Promoting the glycogen synthase, decreasing ammonia accumulation to alleviate exercise-induced central fatigue [52]
**MDA**	An indicator of the extent of lipid oxidation
**SOD, GSH-Px, CAT**	Antioxidant enzymes clearing superoxide radicals to alleviate exercise-induced central fatigue [54]
**NO**	Upregulating the blood flow to ameliorate exercise-induced central fatigue [59, 70]
**nNOS**	Speed limit enzyme of NO synthesis [70]
**Brain glycogn**	Providing energy, and consumption of glycogen influencing monoamine and 5-HT level to promote exercise-induced central fatigue [62, 64, 65]
**Brain lactate**	Increasing excitability of the primary motor cortex to alleviate exercise-induced central fatigue[66, 67]; Activating the GPR81 receptor,aggravating central fatigue caused by exercise[68]
**Ammona**	Possessing deleterious alterations in astrocyte morphology and cerebral energy metabolism to promote exercise-induced central fatigue [71]
**BNDF**	Potentially possessing neuroprotective effect and maintaining energy homeostasis to attenuate exercise-induced central fatigue [72]
**IL-1β**	Potentially influencing DA and 5-HT neurotransmitter systems, hippocampal neuroplasticity and neurogenesis, and hypothalamic pituitary-adrenal axis function to promote exercise-induced central fatigue [73]

### 4.4. Other potential biomarkers

Ammonia, typically deemed a metabolic byproduct of nitrogenous compounds such as amino acids, possesses significant toxicity and can permeate the blood-brain barrier, thereby diminishing cerebral functionality [74]. Elevated blood ammonia concentrations increase the exposure of organs, notably the brain, to the ammonia's deleterious effects. Heightened brain ammonia levels can trigger detrimental changes in astrocyte morphology and cerebral energy metabolism, subsequently impacting neuronal operations [71]. Ammonia levels tend to escalate during diverse exercise types, especially prolonged submaximal activities [8, 75].

Brain-derived neurotrophic factor (BDNF), a neurotrophin intrinsically linked with cognition and learning, is readily modulated by exercise and physical activity [76]. Existing research has probed the nexus between physical activity and BDNF elevation [72], underscoring BDNF's roles in neurotrophy, neuroprotection, and energy homeostasis. However, more conclusive evidence is required in future investigations. Typically, IL-1β remains at subdued concentrations within the central nervous system. Yet, during episodes of acute fatigue, IL-1β levels surge [77]. Moreover, IL-1β can mediate the DA and 5-HT neurotransmitter systems, influence hippocampal neuroplasticity and neurogenesis, and modulate the function of the hypothalamic-pituitary-adrenal axis, potentially leading to the onset of chronic fatigue syndrome [73]. Further inquiries into the correlation between IL-1β and exercise-induced central fatigue are warranted.

## 5. Non-medicinal interventions for alleviating exercise-induced central fatigue

A range of non-medicinal interventions, spanning from caffeine and amino acids to carbohydrate supplements and natural product treatments, are employed to mitigate exercise-induced central fatigue. Both animal studies and clinical trials have affirmed that these interventions effectively postpone central fatigue onset and enhance exercise performance.

### 5.1. Caffeine

As a potent adenosine antagonist, caffeine can significantly curb central adenosine neurotransmission, thereby fostering the release of excitatory neurotransmitters such as DA and NA [78, 79]. When rats are injected with caffeine intraperitoneally, it thwarts the decline in run time to fatigue, heat production, and extracellular DA release instigated by non-selective adenosine receptor agonists. Given that neither caffeine nor these agonists influence NA or 5-HT release, caffeine's ability to enhance endurance and surpass bodily limits may be due to its blockade of adenosine receptors, leading to increased DA release in the brain[80, 81].

Caffeine supplementation markedly elevates the endurance capacity of athletes across various exercise types, particularly during performance troughs [82, 83]. The enhancements seen in well-trained soccer players' performance are predominantly attributable to caffeine's positive impact on the CNS and/or neuromuscular function [84]. Moreover, even in the face of phosphocreatine depletion and H+ accumulation during intensive knee extensor exercises, caffeine can bolster high-intensity exercise tolerance. This is possibly due to an increased central motor drive and corticospinal excitability, linked with afferent feedback from muscle milieu disturbances, resulting in intensified inhibitory input to spinal and supraspinal motor neurons [85]. Additionally, caffeine's potential influence on neuromuscular fatigue is also suggested by physiological parameters like voluntary activation (VA), M wave, and PTw [86]. Furthermore, moderate doses of caffeine can enhance endurance in heated conditions without compromising thermoregulation during extended exercise [87]. Collectively, this evidence underscores caffeine supplementation as a vital strategy to counteract exercise-induced central fatigue. However, this effect is also highly individual and related to a variety of factors, such as genetics, anxiety, habitual caffeine intake, time of caffeine intake, training status, environment, etc. It may also cause side effects such as sleep disturbances and anxiety [88].

### 5.2. Amino acid supplements

Branched-chain amino acids (BCAAs), which primarily comprise leucine, isoleucine, and valine, play pivotal roles in protein synthesis and energy production within skeletal muscles during endurance exercises. When muscles absorb and break down BCAAs during exercise, the levels of free TRP increase, helping it cross the blood-brain barrier. This process boosts the synthesis of 5-HT in the brain, which can lead to earlier central fatigue onset and helps prevent muscle damage [89].

Post-half-ironman triathlon blood analysis of athletes reveals a decline in BCAA levels and an upsurge in the TRP/BCAA ratio. This amplifies 5-HT synthesis in the brain, suggesting that the surge in brain 5-HT may be a factor triggering central fatigue [90]. Recent studies have underscored the efficacy of BCAA intake in primarily counteracting central rather than peripheral fatigue. Evidence includes a more prolonged time to exhaustion in BCAA trials compared to placebos, with no significant variations in myoglobin levels, an indicator of muscle fatigue [91]. Chronic supplementation of BCAAs to adult Wistar rats bolsters their endurance in exhaustive swimming tests. However, excessive BCAA consumption diminishes performance due to hyperammonemia [92], This decline is attributed to cerebral NH3 uptake and accumulation, which modifies brain energy metabolism and neurotransmission, negating BCAAs' beneficial effects on central fatigue [71]. Nonetheless, combining BCAAs with arginine and citrulline enhances athletic performance as observed in randomized controlled trials [10, 93]. Both arginine and citrulline amplify nitric oxide biosynthesis and/or bolster the urea cycle to expel excess NH3, thereby reducing the plasma free TRP/BCAA ratio and attenuating exercise-induced central fatigue [94]. Moreover, BCAA and ornithine aspartate ingestion optimizes reaction times during high-intensity exercises in healthy young men, a benefit linked to ammonia elimination during recovery [95].

Direct supplementation of neurotransmitter amino acids also emerges as a promising strategy to delay exercise-induced central fatigue. GABA, a non-protein amino acid prevalent in both plants and animals, functions as an inhibitory neurotransmitter in mammals. Administering mulberry leaf-derived GABA to male NIH mice notably augments loaded-swimming durations and curtails blood lactate and urea nitrogen concentrations [49]. Glutamine, another amino acid neurotransmitter, counters fatigue by fostering glycogen synthesis and reducing ammonia buildup [96]. Additionally, co-supplementation of glutamine and alanine diminishes muscle ammonia and plasma free fatty acid (FFA) levels, while enhancing hypothalamic 5-HT, the free TRP/total TRP ratio, and the 5-HT/DA ratio, thereby alleviating central fatigue in resistance-trained Wistar rats [97]. Intriguingly, neither glutamine intake alone nor its combined use with alanine significantly boosts physical performance [52, 97], suggesting that heightened 5-HT levels in the brain are not the sole determinants of exercise efficacy.

**Table 2:** Non-medicinal interventions delay the onset of exercise-induced central fatigue.

Intervenitions	Animal model	Human test	Mechanism	Ref
**Caffeine**	Male Wistar rats with treadmill fatigue	Young male with single-leg knee extensor exercise	Blocking adenosine receptor to increase DA release Disturbing afferent feedback of the muscle milieu	[80, 85]
**BCAA**	Male Wistar rats with swimming exercise	Male long-distance runners with an incremental exercise	Enhancing TRP concerntration and facilitating it crossing through blood-brain barrier to synthesize more 5-HT	[89, 92]
**BCAA combined with arginine and citrulline**	NA	Male and female swimmers with different swimming test; Male taekwondo athletes with high-intensity intermittent exercise;	Arginine and citrulline can increase nitric oxide biosynthesis and/or urea cycle to remove excess NH_3_ induced by BCAA supplementation.	[10, 93]
**BCAA and ornithine aspartate**	NA	Endurance-trained men with submaximal cycloergometer exercise	Eliminating ammonia induced by BCAA supplementation.	[95]
**GABA**	Male NIH mice with loaded-swimming exercise	NA	Serving as an inhibitory neurotransmitter	[49]
**Glutamine and Glutamine with alanine**	Male Wistar rats climbing a vertical ladder with a load	NA	Promoting glycogen synthesis and decreasing ammonia accumulation; increasing hypothalamic 5-HT, free TRP/total TRP ratio, and the 5-HT/DA ratio	[52, 97]
**CHO supplements**		Male runners with different quantity or quality of CHO	Promoting CHO oxidation and insulin response	[102]
**Zena F-III**	Male ddY mice walk using a programmed motor-driven wheel cage	NA	Decreasing calcium and DA levels in the brain	[106]
**Morinda citrifolia leaves**	Female ICR mice with weight-loaded swimming test	NA	Possessing antioxidant, anti-inflammatory activity, as well as improving neurotransmitters expressions, transporter or receptor levels	[107]
**Macamides**	Balb/c mice with swimming test	NA	Decreasing MDA contents and increasing SOD and GSH-PX activities in the brain	[108]
**polysaccharide from Spirulina**	Male Sprague-Dawley rats with treadmill running	NA	Suppressing the exercise-induced enhancement of 5-HT and TPH2 expression and preventing the exercise-induced decrease of 5-HT1B expression in the caudate putamen of exercised rats	[39]
**Leucine and glycine dipeptides**	Male ICR mice with treadmill exercise	NA	Enhancing the levels of DA, BDNF and phosphorylated-extracellular signal-regulated kinase in the brains	[111]
**Colostrum serum**	Male Sprague-Dawley rats with treadmill exercise	NA	Suppressing exercise-induced expression of TPH and 5-HT, 5-HT_1A_ and 5-HTT expression in the dorsal raphe nuclei	[112]

### 5.3. Carbonhydrates supplements

Carbohydrates (CHO) serve as a vital energy source for humans and significantly influence exercise. By maintaining blood glucose levels, carbohydrate intake curtails the onset of central fatigue. Furthermore, carbohydrates are integral to the central nervous system's energy metabolism [98]. The impact of CHO consumption on mitigating central fatigue is influenced by exercise duration, type of exercise, hypoxia levels, altitude, and individual metabolic rates [99]. Recent studies have highlighted that CHO mouthwash can enhance exercise performance across various activities, likely due to the oral perception of CHO [100, 101]. More so, numerous investigations reveal that well-trained male runners, when on a high-CHO diet compared to a low-CHO one, demonstrate superior maximal voluntary contraction, voluntary activation, central activation rate, and heightened CHO oxidation and insulin responses. These findings emphasize CHO supplementation's positive effects in mitigating exercise-induced central fatigue [102]. The combination of protein and carbohydrate intake is more effective in delaying central fatigue and can also increase repetitive exercise ability [103]. Nonetheless, CHO intake for enhancing exercise capacity and reducing central fatigue still poses challenges. Potential risks include choking from carbohydrate mouthwash [101], and gastrointestinal strain due to CHO intake during exercise, potentially hampering performance. The methodologies for CHO supplementation and its measurement lack clear guidelines, presenting avenues for future research [104].

### 5.4. Natural products

While numerous natural products reportedly offer anti-fatigue benefits [105], those specifically related to counteracting exercise-induced central fatigue remain relatively scarce. Notable examples encompass health care products such as Zena F-III [106], water extracts of Morinda citrifolia leaves [107], the Lepidium meyenii Walp-derived compound macamides [108], and polysaccharides from Spirulina [39]. Moreover, various plant components, including ginseng, rhodiola rosea, and robinia pseudoacacia, have shown promise in alleviating exercise-induced central fatigue [109]. Additionally, derivatives from natural entities, such as citrus essential oil (CEO), manifest beneficial effects on central fatigue [110]. In this review, we summarize non-pharmacological interventions related to exercise-induced central fatigue (Table 2).

Zena F-III is a liquid nutritive and tonic product, popular in Japan, derived from the formulations of Chinese herbal medicine. When orally administered, Zena F-III notably diminishes the elevated calcium and DA levels in the brains of male ddY mice subjected to forced walking. This suggests its potential protective effect against exercise-induced central fatigue [106].

The Morinda citrifolia fruit, traditionally used by Polynesians to tackle fatigue and various ailments, has been validated for its effectiveness in improving endurance in both mice and clinical trials. However, recent studies indicate that the water extract from Morinda citrifolia leaves surpasses the fruit in enhancing performance, as observed in a weight-loaded swimming animal model. This extract, enriched with scopoletin and epicatechin, showcases remarkable anti-oxidative and anti-inflammatory properties. Furthermore, it aids in optimizing neurotransmitters (DA, NA, 5-HT) expressions, and transporter or receptor levels, subsequently aiding in delaying fatigue onset during physical exertion [107].

Macamides (N-benzyloleamide) are extracted from Lepidium meyenii Walp. (commonly known as maca), a substance traditionally consumed in Peru to boost vitality. Research exploring the effects of macamides on endurance has unveiled its pronounced anti-fatigue attributes. In prolonged swimming tests conducted on mice, macamides reduced MDA contents and amplified SOD and GSH-PX activities in the brain [108].

Spirulina, a single-celled blue-green algae utilized as a nutritional supplement, contains polysaccharides that effectively delay fatigue onset in rats subjected to treadmill exercises. Besides increasing hemoglobin levels, these polysaccharides also reduce blood lactic acid, urea nitrogen, and creatine kinase levels. Moreover, the polysaccharide extract from Spirulina platensis counters the exercise-induced surge of 5-HT and TPH2 expression and prevents the decline of 5-HT1B expression observed in the caudate putamen of exercised rats [39].

### 5.5. Other interventions

Dipeptide supplements are increasingly recognized for their role in counteracting exercise-induced central fatigue. Particularly, the dipeptides leucine-glycine and glycine-leucine, which are prominently found in Danish porcine placenta, are effective in elevating DA, BDNF, and phosphorylated-extracellular signal-regulated kinase levels in the brains of Male ICR mice exposed to exhaustive treadmill exercise [111].

Colostrum, the initial milk secreted by mothers during the first 72 hours post-birth, is rich in essential nutrients and bioactive compounds. It plays a pivotal role in protecting against pathogenic microbes, fostering immature intestinal development, supporting organ and tissue growth, and bolstering the immune system in newborns. Current research indicates that colostrum serum can effectively inhibit the exercise-induced expression of TPH and 5-HT, as well as regulate 5-HT1A and 5-HT transporter (5-HTT) expression in the dorsal raphe nuclei of male Sprague-Dawley rats [112].

Additionally, certain non-invasive methods can help prevent or alleviate central fatigue. Transcranial direct current stimulation (tDCS) enhances cortical excitability and shows promise in combating supraspinal fatigue. Thus, tDCS presents itself as a viable approach to mitigating exercise-induced central fatigue and enhancing athletic performance [113]. However, the effect of tDCS on corticospinal excitability varies greatly among individuals, which may be the result of individual anatomical differences. In addition, some studies have shown that the controversy about the effectiveness of tDCS in enhancing function may also be caused by the difference in the electrode montage used in the experiment (i.e., the lateral montage and the external montage). Placing the cathode electrode on the shoulder may be more effective than placing it on the dorsolateral prefrontal cortex [114]. Cold water immersion (CWI) performed immediately after a simulated football game significantly reduced the central and peripheral components of fatigue, resulting in faster recovery of neuromuscular function and performance indicators, and reduced central and peripheral fatigue. Therefore, CWI can serve as a recovery strategy for athletes when the competition schedule is more intensive [115, 116]. Low-resistance bicycle training is effective, beneficial, and feasible for enhancing knee extensor strength and peripheral strength in early Parkinson's disease patients, particularly those experiencing noticeable central fatigue [117].

## 6. Conclusions

Central fatigue's pronounced link to endurance and overall exercise performance has garnered significant interest from both the scientific community and athletes. Implementing effective and healthy strategies to mitigate exercise-induced central fatigue is paramount for prolonging athletes' careers. During physical exertion, peripheral signals relayed to the CNS by group III/IV muscle afferents activate specific brain regions, potentially triggering central fatigue, which subsequently influences muscular contractions. Biomarkers indicative of this fatigue encompass neurotransmitters and their regulators, molecules tied to brain oxidative processes, brain glycogen, its byproduct lactate, and other potential indicators. Interventions, ranging from caffeine and amino acids to carbohydrates and various non-medicinal natural products, have demonstrated efficacy in delaying the onset of such fatigue.

In this review, our focus is primarily on non-medicinal interventions with proven efficacy against exercise-induced central fatigue. Further investigations are essential to determine if other treatments, reported to enhance physical performance, act centrally to alleviate fatigue. Additionally, individual differences in exercise-induced central fatigue are significant, and strict control of a single variable is challenging. Due to the limited sample size of the experiment, it is difficult to obtain specific differences between different genders and ages. Consequently, further research is needed for some issues. Firstly, current measurement techniques for central fatigue struggle to accurately identify specific active regions of the cerebral cortex. Most of the measurement methods measure the degree of central fatigue caused by exercise indirectly by first measuring some relevant indicators, so more direct and accurate strategies and technologies are in great requirement to measure the central fatigue and to observe the active regions in the brain during exercise. Second, some potential biomarkers, such as BNDF and IL-1β, need more evidence to determine their role in exercise-induced central fatigue. The double-sided action mechanism of lactic acid on exercise-induced central fatigue also needs further study and discussion. Lastly, the effectiveness of numerous non-drug interventions remains uncertain and is influenced by various factors. For instance, the use of carbohydrate mouthwash does not universally decrease exercise-induced central fatigue and might be linked to muscle glycogen availability and oxygen levels in the environment [118, 119], indicating some potential factors such as the quality and quantity of CHO, exercise type, gender differences, and the mode of carbohydrate intake, may influence outcomes [99], the effect of caffeine on reducing central fatigue caused by exercise is also affected by a variety of factors, and can also cause other side effects and so on. Therefore, future studies should prioritize resolving these issues, increasing the sample size of studies, delineating gender and age disparities, and devising more precise methods to measure exercise-induced central fatigue. Concurrently, effective non-drug interventions for exercise-induced central fatigue should identify optimal methods and measurements while minimizing potential risks of adverse effects.

## Data Availability

Data availability is not applicable to this article as no new data were created or analyzed in this study.
